# Diel activity of newly metamorphosed juvenile sea lamprey (*Petromyzon marinus*)

**DOI:** 10.1371/journal.pone.0211687

**Published:** 2019-02-06

**Authors:** Scott M. Miehls, Christopher M. Holbrook, J. Ellen Marsden

**Affiliations:** 1 U.S. Geological Survey, Great Lakes Science Center, Hammond Bay Biological Station, Millersburg, MI, United States of America; 2 Rubenstein Ecosystem Science Laboratory, University of Vermont, Burlington, VT, United States of America; Laboratoire de Biologie du Développement de Villefranche-sur-Mer, FRANCE

## Abstract

Timing of activity, especially for juvenile anadromous fishes undertaking long migrations can be critical for survival. River-resident larval sea lamprey metamorphose into juveniles and migrate from their larval stream habitats in fall through spring, but diel timing of this migratory behavior is not well understood. Diel activity was determined for newly metamorphosed sea lamprey using day/night net sampling and passive integrated transponder (PIT) telemetry in two natural streams and PIT telemetry in an artificial stream. Downstream migration was primarily nocturnal in all studies. All but one of 372 sea lamprey were captured during night sampling in the day/night net collections and all detections (N = 56) for the in-stream PIT telemetry occurred within a few hours after sunset. Most (81% of 48) tagged lamprey moved downstream during the first night following release and moved at speeds consistent with observed water velocities. During long-term observation of behavior in the artificial stream most sea lamprey movement occurred during the night with limited occurrence of movement during daylight hours. Understanding seasonal and diel timing of downstream migration behavior may allow more effective management of sea lamprey for both conservation and control.

## Introduction

Animals use environmental cues to ensure that migrations occur when conditions are most favorable for survival, growth, or reproduction [1]. For juvenile anadromous fishes, timing of downstream migration from tributary rearing habitats to marine or lacustrine feeding habitats may have evolved in response to predation risk, food availability, and abiotic environmental conditions. For example, nocturnal migration is thought to be a predator avoidance behavior common in many fish species [2], but the propensity to migrate at night can vary within and among species [3]. Knowledge of migration timing has the potential to inform conservation of imperiled or threatened populations, but might also be used to improve assessment and control of pests. For example, appropriately timed spills of water and turbine shutdowns at hydropower facilities may limit mortality of downstream migrants such as salmonids [4] or eels [5].

Sea lamprey (*Petromyzon marinus*) are threatened or endangered in a large portion of their native range, while at the same time they are considered an invasive nuisance in the Laurentian Great Lakes of North America. Management strategies in both of these scenarios have targeted the migratory, spawning adult and larval life stages. Where sea lamprey are native, migratory barriers preventing access to spawning grounds are a major impediment to recovery for imperiled lamprey populations, so management practices focus largely on barrier removal and improvements in passage that allow access to spawning habitats [6]. Recently, hatcheries have begun rearing larval lampreys from eggs to bolster diminished populations (*Lampetra fluviatilis*, [7]; *Entosphenus tridentatus* [8]). Where sea lamprey are invasive, strategies to suppress populations currently rely on migratory barriers to prevent adults from reaching spawning sites and limit the area of infestation while simultaneously killing larvae in stream reaches where spawning is successful [9]. In both management scenarios, few efforts have focused on the recently metamorphosed life stage, where better understanding of behavior during a critical life stage transition may reveal opportunities for both conservation [10] and control [11].

Sea lamprey have a complex life cycle, undertaking two migrations and substantial habitat shifts. Larval sea lamprey live in the substrate of freshwater streams for 2 to 12 years before metamorphosing into their juvenile parasitic form [12, 13]. Metamorphosis is generally synchronous within a stream population for those lamprey attaining a minimum length and condition each year [14]; timing and size at initiation of metamorphosis varies among streams, latitudes, and years [15]. After metamorphosis, sea lamprey leave the natal stream and move to juvenile feeding environments, either lakes or oceans [16, 17]. The parasitic phase lasts 12–15 months for the landlocked sea lamprey in the Laurentian Great Lakes of North America [12] and extends beyond two years for anadromous sea lamprey [13] before they return to rivers to spawn and then die.

The migratory period for newly metamorphosed lamprey is protracted compared with the more commonly studied salmonid species (e.g., Atlantic salmon *Salmo salar* [18]; coho salmon *Oncorynchus kisutch* [19]). Juvenile sea lamprey migration typically begins in October and continues through April [12, 20] or May [21]. Prolonged out-migration may buffer sea lamprey populations from environmental perturbations [22, 23], reduce crowding that could attract predators, or be an adaptive trait to prevent all parasitic juveniles from reaching the open-water feeding environment at the same time [13].

Environmental cues such as flow conditions and water temperature influence the onset of outmigration for lamprey [12, 24–25]. Outmigration demonstrably coincides with periods of increased discharge, typically peaking during autumn and spring [13, 16, 26], and the number and magnitude of high-water events during autumn may dictate the magnitude of autumn versus spring migrations [24, 26]. The relationship between downstream movement and water temperature is less clearly defined, but decreasing temperature or cold-water conditions appear important for initiation of downstream movement [13, 27]. To date, little has been published to describe the influence of the day–night cycle on metamorphosed juvenile sea lamprey activity or diel patterns for downstream migration. However, other lamprey species seem to exhibit nocturnal activity patterns [26, 28, 29], suggesting that such patterns could be present in sea lamprey. Additionally, sea lamprey exhibit a negative phototactic response at multiple life stages [30] and are mostly nocturnal as both ammocoetes [31] and migratory adults [32].

We hypothesized that juvenile sea lamprey would be most active and most likely to undergo downstream migration during periods of darkness. To test that hypothesis we conducted three experiments. First, as a proof-of-concept, we collected downstream migrating juvenile sea lamprey continuously for nine days from a natural stream to determine if downstream movement differed between day and night (Study I). We then monitored downstream movement of passive integrated transponder (PIT)-tagged sea lamprey for 42 days in a natural stream to determine timing of downstream movement (Study II) and in an artificial stream under controlled conditions for 88 days to determine daily activity patterns (Study III).

## Methods

### Study I: Day vs. Night sampling

To determine whether downstream-migrating juvenile sea lamprey were more active at night than during the day, we collected sea lamprey at the outflow of the Little Carp River, Michigan (46°50'6.34"N 88°28'57.58"W). The Little Carp River is a small, intermittent tributary to Lake Superior. Two fyke nets (1.22m x 0.91m mouth; 4.76 mm delta mesh), deployed side-by-side, were used to sample the entire outflow where the Little Carp River emptied into Lake Superior. Nets were emptied at sunrise and sunset daily from November 1, 2011 until November 9, 2011. All sea lamprey were removed and counted and other species were immediately released. Water temperature was recorded at 15-min intervals using an Onset HOBO U22 temperature logger and daily averages were calculated. Collections were permitted by the State of Michigan under the Michigan Department of Natural Resources Scientific Collectors Permit issued to U.S. Geological Survey, Hammond Bay Biological Station December 12, 2007; amended February 23, 2011.

### Study II: Instream telemetry

To determine timing of downstream movement we monitored downstream passage for newly metamorphosed juvenile sea lamprey using PIT telemetry November 3, 2014 –December 15, 2014. Downstream-moving newly-metamorphosed juvenile sea lamprey (n = 290) were initially collected using a combination of drift nets (0.61 m x 0.30 m mouth; 4.8 mm delta mesh) and fyke nets (1.83 m x 0.91 m mouth; 6.4 mm delta mesh main body; 4.8 mm delta mesh cod end) deployed near the confluence of Morpion Stream and Pike River, Quebec (45°10'23.68"N 73° 2'16.98"W) as part of a spatial distribution study [33]. Below the confluence, the Pike River drains into Missisquoi Bay at the northern end of Lake Champlain. Nets were emptied daily and all fish were counted. Juvenile sea lamprey were retained for tagging while all other species were counted and released immediately. Surgical procedures followed those described in [34]. Lamprey were anesthetized by immersion in a 0.026 mL/L concentration of AQUI-S 20E (AQUI-S, New Zealand) until unresponsive. A sterile scalpel was used to make a 2–3-mm incision on the left lateral side approximately 20 mm posterior to the gill pores. A 12-mm HDX PIT tag (12 mm × 2.12 mm, 83 mg, Oregon RFID) was inserted by hand and guided posteriorly into the body cavity away from the incision. Tagged lamprey were placed in an aerated bucket with fresh water until they became mobile and then transferred to in-stream livecars. Sea lamprey were held for a minimum of two days of observation and then released approximately 6 km upstream from the river mouth, 5.6 km above the traps. Lamprey were released during late morning once net tending was completed. Live-cars were tipped to allow lamprey to swim freely; most would immediately seek shelter or burrow. Collections from Morpion Stream, Quebec during 2014 were conducted under Québec Ministère des Resources Naturelles permis à des fins scientifiques 2012-10-01-1436-16-SP to Ellen Marsden and a scientific collection permit from the Vermont Department of Fish and Wildlife. Fish care and protocols for fish holding, surgery, and tagging were conducted under University of Vermont Institutional Animal Care and Use Committee protocol 13–017.

PIT antenna arrays were constructed 4.8, 5.0, and 5.2 km downstream of the release point. Each antenna was a loop of 6 gauge copper wire, 0.5 m high; four antennas were installed per array, each spanning the width of the river, and stacked vertically to encompass the entire water column from the substrate up to 2 m depth. Each antenna array was energized and PIT tag detections consisting of tag ID number, date, and time were collected using an Oregon RFID multiple antenna HDX reader powered with 12 V deep cycle batteries. Detection probability (*p*) was estimated as the proportion of tagged sea lamprey captured in downstream nets that were detected at one or more upstream PIT antennas. A 95% confidence interval was calculated using Clopper-Pearson exact method [35].

### Study III: Artificial stream

**Fig 1:**
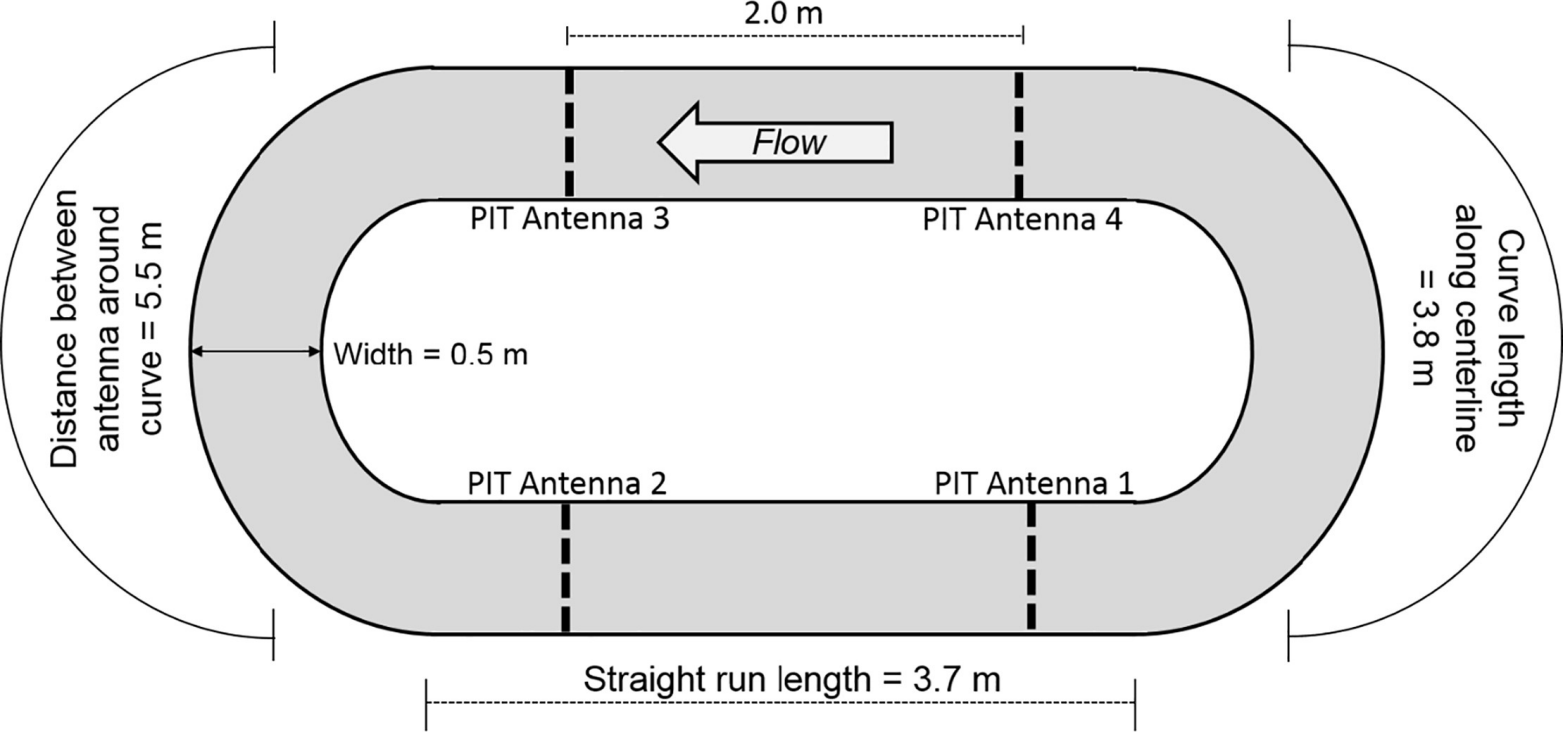
Study III artificial stream schematic. Upstream and downstream movement of newly-metamorphosed juvenile sea lamprey was monitored in a 15-m circumference artificial stream during November 6, 2014 –February 2, 2015.

To determine individual daily activity patterns, we monitored movement of newly metamorphosed juvenile sea lamprey in an artificial stream using PIT telemetry (Fig 1). The artificial stream was oval, with a 15 m circumference at the channel centerline, and a 0.5 m wide channel. Sand/gravel substrate was added to a depth of 0.05–0.10 m, and water depth was maintained at 0.5 m. PIT antennas were positioned 0.85 m from the beginning and end of each straight section (4 antennas total) and completely encircled the raceway channel. Detection range for each antenna was approximately 0.3 m upstream and downstream. Stream sediment was replaced with compacted gravel in the detection zone around each antenna to prevent lamprey from burrowing and thus prevent detection while burrowed. The raceway was supplied with ambient water from Lake Huron and flow was maintained at 0.10–0.12 m·s^-1^ (measured at 60% depth at midpoint of the straight stream sections) using two recirculation pumps positioned in each curve at opposite ends of the stream.

Forty-eight newly-metamorphosed juvenile sea lamprey (mean length = 164.5 ± 9.1 *SD* mm; mean weight = 6.3 ± 1.1 *SD* g) were implanted with 12 mm HDX PIT tags October 23, 2014, held four days in two 38-L aquaria for observation, and stocked into the artificial stream October 27, 2014. Sea lamprey were removed November 2, 2014, to inspect condition and tag retention after being allowed 5 days to burrow into the stream sediment. All sea lamprey were returned to the stream and allowed 4 days to acclimate; PIT detection recording and analysis began at 1200 (UTC– 0500 hr) November 6, 2014 and continued uninterrupted until February 2, 2015 at which point icing prevented continued operation of the stream. PIT antennas were tested daily using a test tag and detection records were downloaded weekly. Water temperature was recorded at 10-min intervals throughout the observation period using an Onset HOBO U22 temperature logger. The stream was checked daily between 1000 and 1200 (UTC –0500 hr) for mortalities. Prey were not provided for juvenile sea lamprey in the artificial stream.

An Oregon RFID multiple antenna HDX reader recorded the history of detection events for each sea lamprey, where each detection event was comprised of one or more contiguous detections on each antenna. We defined movement events as transitions among antennas. Movements were bounded by the first detection on one antenna and the first detection on another antenna to reduce bias in movement rate calculations due to size of detection range. Antenna detection range was tuned to be equal among antennas and therefore assumed equal for analysis. Direction (‘upstream’, ‘downstream’, or ‘unknown’), longitudinal distance moved (i.e., between antennas along centerline), and diel period (‘day’ or ‘night’) were assigned to each movement event. Direction, based on water flow direction, was only known for movements among adjacent antennas (‘first-order transitions’) and unknown for nonsequential detections. The proportion of first-order transitions was interpreted as a coarse measure of detection efficiency.

False detections can be present in PIT data for several reasons (e.g., tag signal collisions, antenna ‘cross-talk’ or ‘bleedover’, etc.) therefore we applied several filters to the detection and movement data. First, we omitted all detection events for an individual tag with overlapping timestamps at multiple antennas, i.e., when a lamprey appeared to be two places at one time. We then removed movement events during which movement rate exceeded 1 m·s^-1^. Movement greater than 1 m·s^-1^ was likely not possible given maximum swimming speeds observed for juvenile lamprey (5.2 body lengths·s^-1^; [36]) and the constant water velocity maintained in the artificial stream (0.10–0.12 m·s^-1^). Finally, to ensure that analyses reflected active swimming we removed transitions that took more than three minutes, indicating the lamprey ceased moving and rested between antennas.

Movements were grouped into four classes by a combination of direction and diel condition (e.g., downstream-day, upstream-day, downstream-night, upstream-night) and a Kruskal-Wallis rank sum test [37] was used to determine if the proportion of movements that occurred in any class differed among fish. Differences between specific groups were tested using Nemenyi's non-parametric all-pairs comparison test, which was performed using the ‘kwAllPairsNemenyiTest’ function using the Tukey distribution in the R package ‘PMCMRplus’ [38]. A siginificance level of 0.05 was used for all tests.

## Results

### Study I: Day vs. Night sampling

**Fig 2:**
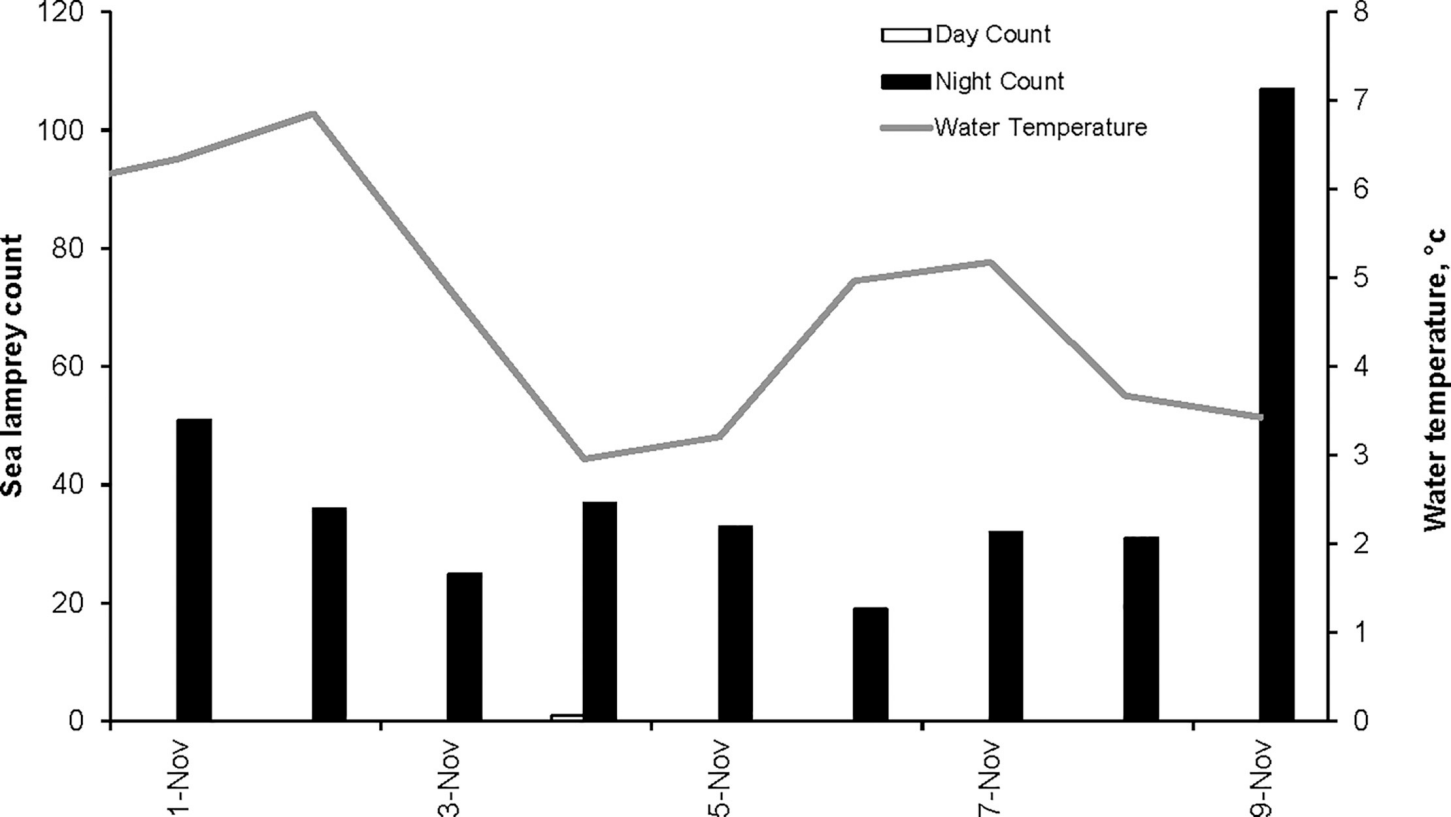
Study I: Diel sampling for downstream migrating juvenile sea lamprey. Newly metamorphosed juvenile sea lamprey were captured using fyke nets at the mouth of Little Carp River, Michigan, fished from sunrise to sunset (grey bars) and sunset to sunrise (black bars) during November 1, 2011 –November 9, 2011. A total of 372 sea lamprey was captured of which 371 were captured during nighttime and one was captured during daytime (asterisk; 11/4/2011). Mean daily water temperature (grey line; secondary y-axis) recorded at 15 min intervals and averaged for each 24-hr period beginning at 00:00 hr.

Among 372 juvenile sea lamprey captured, 371 were collected during nighttime and one was collected during daytime (Fig 2). Counts per 24-hr period averaged 33 ± 9.4 (*SD*) sea lamprey during the first 8 days of sampling; 107 sea lamprey were captured during the evening of November 9, 2011, coinciding with a high water event. Water temperature decreased during the 9-day period from 6.0°C on November 1 to 2.1°C on November 9, 2011 (Fig 2).

### Study II: Instream telemetry

Detection probability for a tagged sea lamprey moving past all downstream detection locations was 35% (95% C.I: 22–50%). Forty-one (14%) of the tagged sea lamprey were detected by at least one antenna array during their first downstream transit following tagging and release. Forty-eight tagged sea lamprey were recaptured in the downstream fyke nets and released a second time at the upstream release point; seven (15%) of these individuals were detected moving back downstream during subsequent transits. The difference between estimated detection probability and percent of released lamprey detected may have resulted from mortality, halted migration between release and PIT arrays, or environmental factors causing detection probability to be correlated with likelihood of capture in downstream fyke nets. For example, the detection probability would be over-estimated if lamprey were both less likely to be detected and less likely to be caught during high flow conditions.

**Fig 3:**
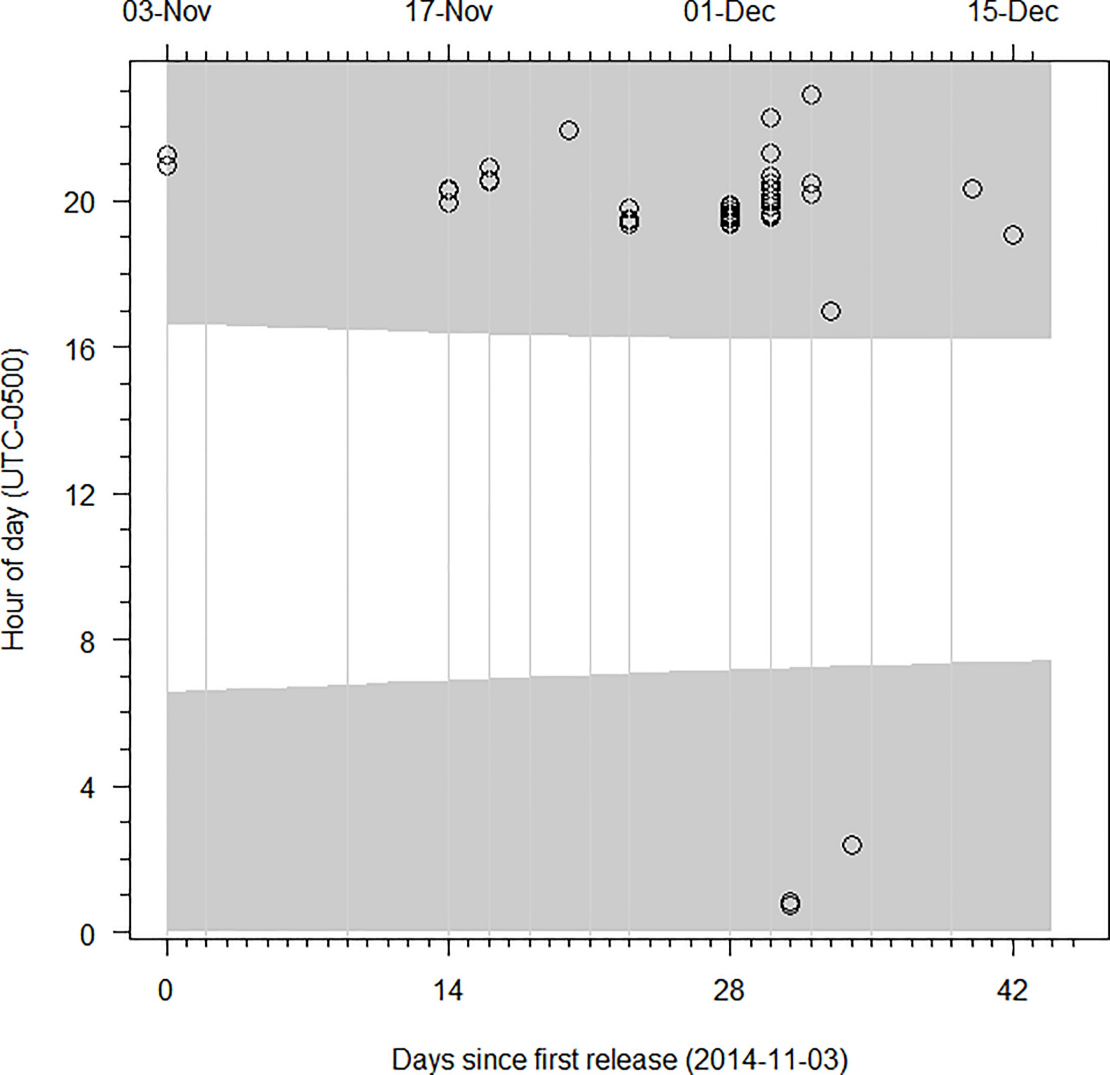
Study II: Timing of downstream movement by juvenile sea lamprey. Detections of PIT implanted juvenile sea lamprey (circles) are shown by time of day (y-axis) for individuals that moved downstream past PIT antenna arrays installed in Morpion Stream, Quebec. Groups of lamprey were tagged and released approximately 5 km upstream of antenna arrays throughout the observation period November 3, 2014 –December 17, 2014. Shading indicates period between sunset and sunrise. Vertical grey lines indicate release events.

All detections occurred after dark, with 91% occurring between 1900 and 2200 (UTC –0500 hr; Fig 3). Eighty-one percent of detections occurred during the first night immediately following release. Five sea lamprey were detected at both antenna array 3 and array 2 while moving downstream and seven sea lamprey were detected at both array 2 and array 1; transit time between detection points for all of these individuals ranged 4–5 min. Using the observed transit times for these 12 sea lamprey relative to distances between arrays (~200 m), approximate movement velocity was calculated between 0.67 and 0.83 m·s^-1^, well within the range of water velocities observed for Morpion Stream. At those velocities, tagged sea lamprey could have moved from the release point to the PIT arrays (a distance of 4,800–5,200 m) in 1.6–2.2 hr by simply drifting with the current. Given that sea lamprey first appeared at the PIT arrays three or more hours after dark, this observation suggests that downstream movement was likely initiated after dark.

### Study III: Artificial stream

Water temperature was 8.4°C at the beginning of the observation period and dropped to < 6°C by November 15 and < 4°C by November 27 (9 days and 23 days after the observation period began). Tag retention was 100% and survival was 98% (47 of 48) for the duration of the study. The single mortality occurred 60 d into the observation period; the sea lamprey showed signs of disorientation and lethargy during a daily check on January 5, 2015 and was removed from the stream. Data from the sea lamprey that died were omitted from all movement analyses because we did not know when it became impaired.

Among 102,893 detection events that occurred during the study period, 14,345 (13.9%) had overlapping timestamps and were removed. The remaining detections were distilled into 22,371 movement events, of which 20,735 were first-order transitions (i.e., 92.7% detection efficiency). We removed 836 movement events (3.7%) during which movement rate exceeded 1 m·s^-1^. Finally, we removed 795 movement events (3.7%) as a result of the 3-min transit time threshold, with deleted transit times ranging from 3.0 min to 82.1 days (median 9.5 min).

**Fig 4:**
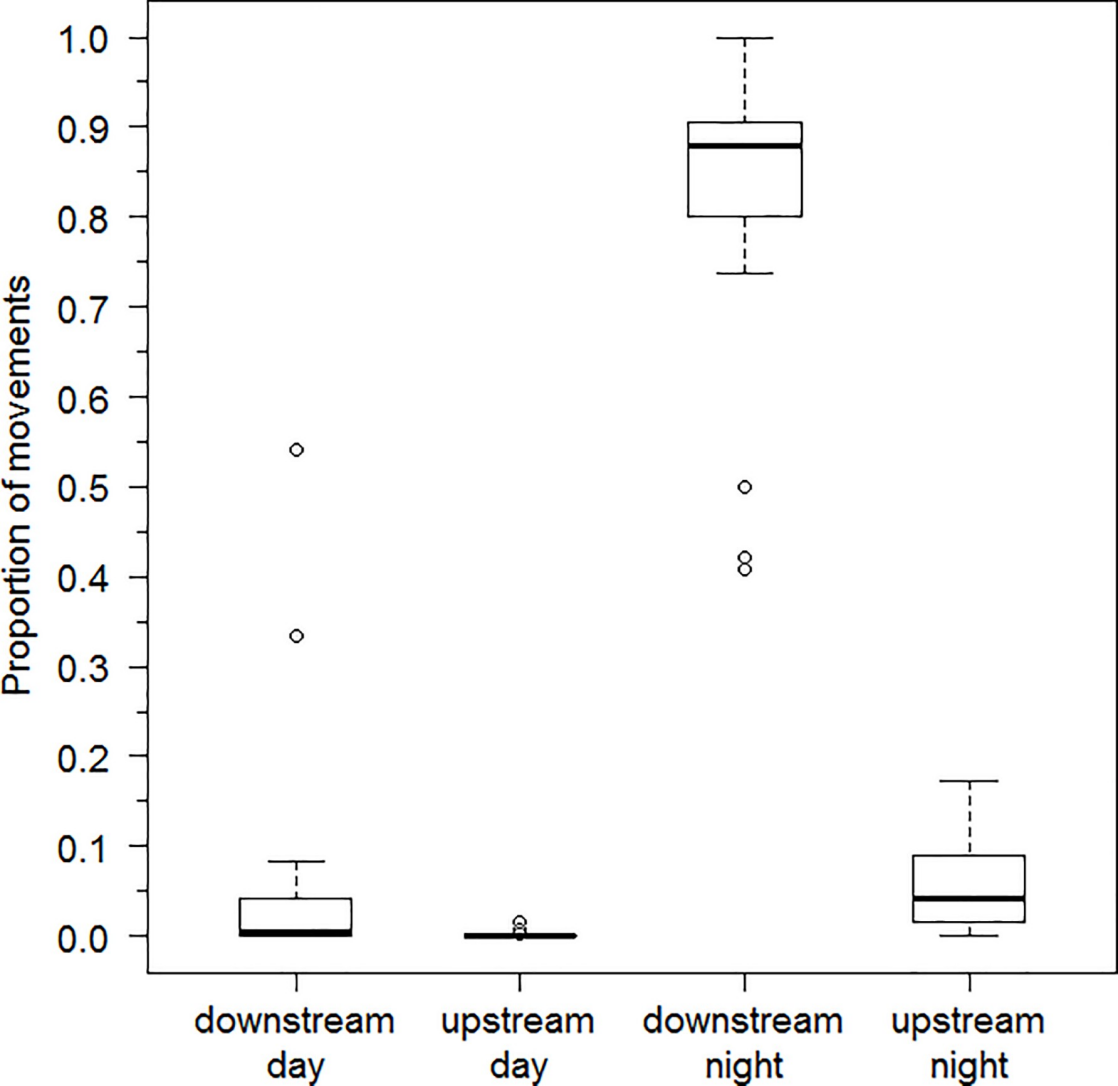
Study III: Direction and timing of movement by juvenile sea lamprey in an artificial stream. Proportion of total movements by newly metamorphosed juvenile sea lamprey (N = 25) that occurred in either the downstream or upstream direction during day or night over a 91 day observation period in an artificial stream, November 6, 2014 –February 2, 2015. Solid horizontal lines show median proportion among fish, boxes show first and third quartiles, whiskers show 1.5 times interquartile range (IQR) from median, and symbols show observations greater than 1.5 IQR from median.

**Fig 5:**
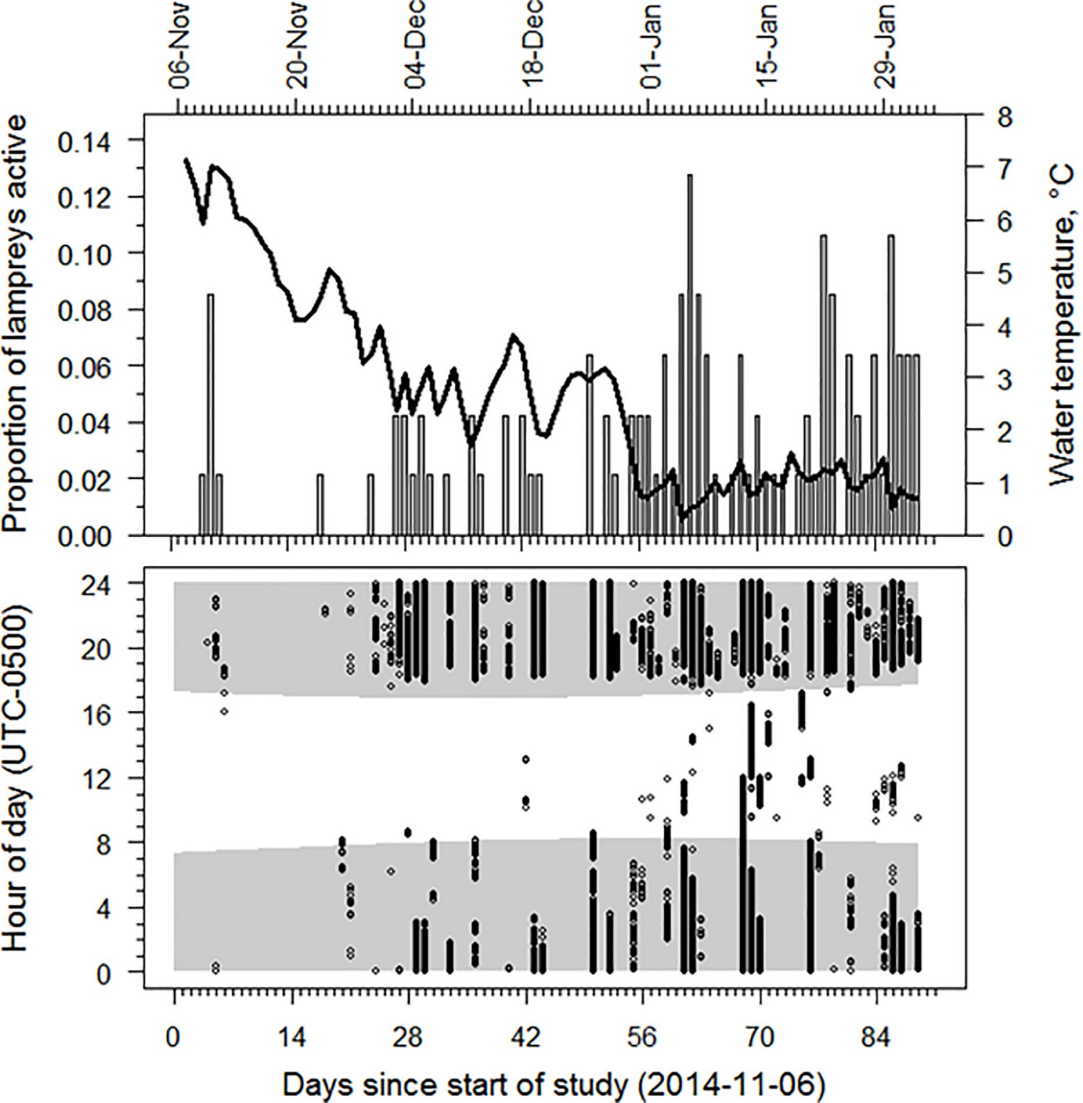
Study III: Downstream movement activity by juvenile sea lamprey in an artificial stream. Proportion of newly metamorphosed juvenile sea lamprey that moved downstream at least once (vertical bars) each day, with mean daily water temperature (black line; secondary y-axis), in an artificial stream November 6, 2014 –February 2, 2015 (Panel A); timing of diel movement events during the 91-day observation period (Panel B). Shading in indicates period between sunrise and sunset.

**Fig 6:**
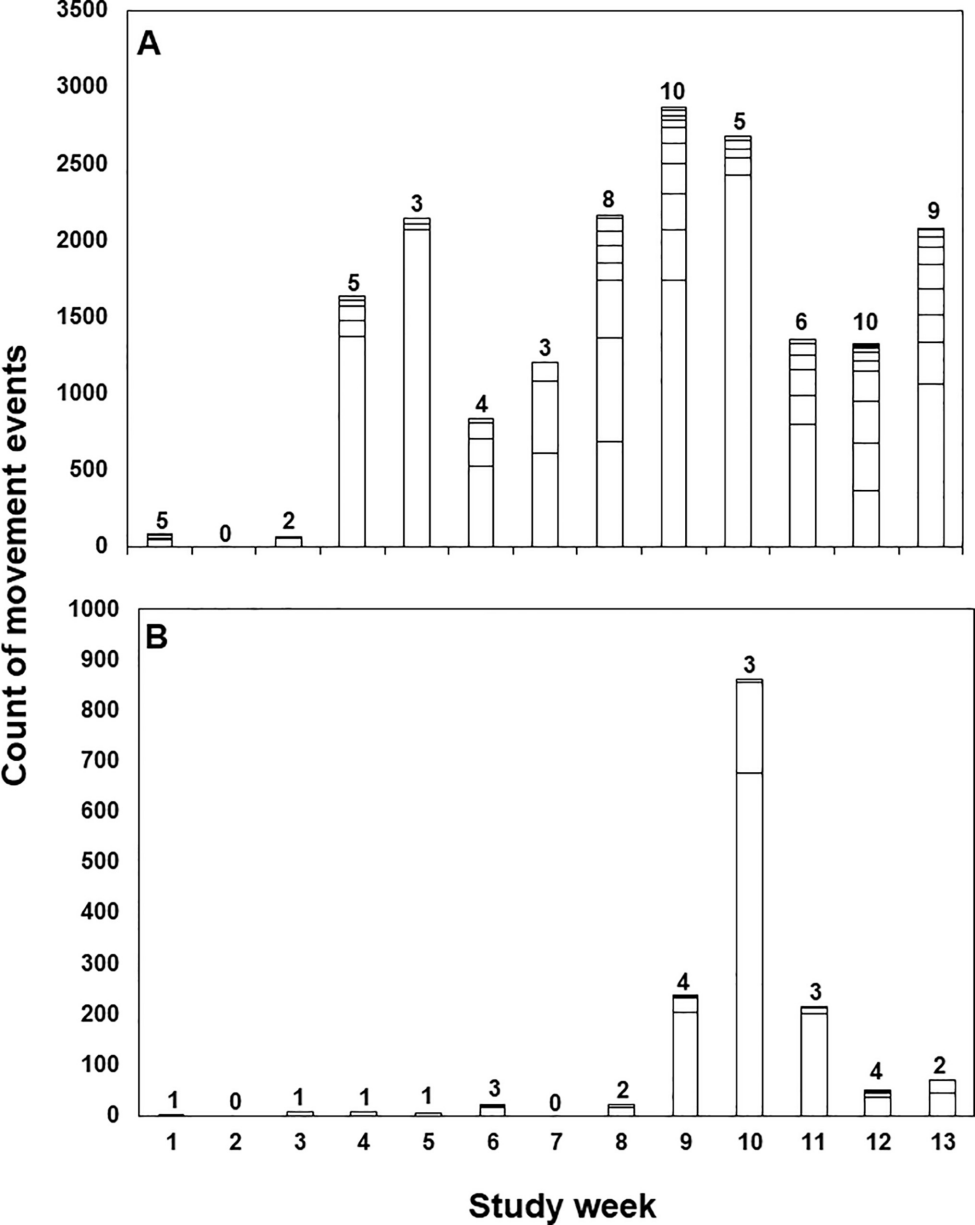
Study III: Weekly counts of downstream movement events for juvenile sea lamprey in an artificial stream during day and night. Weekly count of downstream movement events (vertical bars) detected during hours of dark (Panel A) and during daylight hours (Panel B) for 48 PIT tagged juvenile sea lamprey in an artificial stream November 6, 2014 –February, 2, 2015. Counts for individual lamprey are represented by individual bars within each stacked column and the count of lamprey detected per week is provided above each column. Over the duration of the observation period, 14 individual lamprey were detected moving during daylight hours and 25 individual lamprey were detected moving during dark hours.

**Fig 7:**
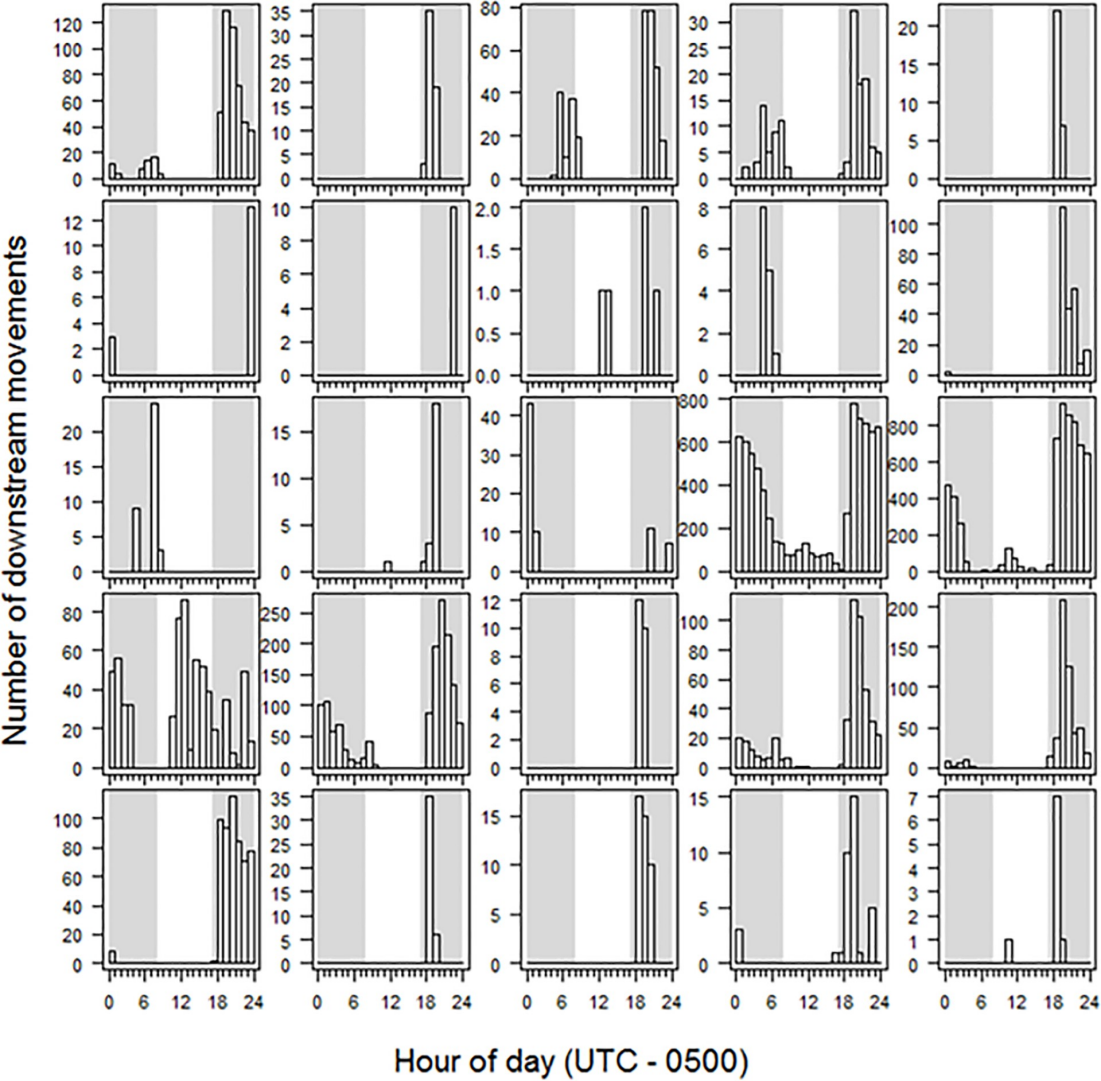
Study III: Individual downstream movement activity for juvenile sea lamprey in an artificial stream. Number of downstream movements (vertical bars) detected during each hour of the day for each of 25 tagged juvenile sea lamprey (one per plot) in an artificial stream November 6, 2014 –February, 2, 2015. Shading indicates nominal day/night hours (delineated by median sunrise and sunset).

Twenty-five of the 47 tagged sea lamprey (53%) actively left shelter at least once during the course of the study with the number of movement events per lamprey ranging from 6 to 8,304 (median 66 movement events). Among lampreys that moved (N = 25). 40.9–100% (median 87.9%) of movements were downstream at night, 0–17.2% (median 4.1%) of movements were upstream at night, 0–54.2% (median 0.6%) of movements were downstream during daylight, and 0–1.5% (median 0%) were upstream during daylight (Fig 4). The proportion of movements that occurred at night in the downstream direction was significantly larger (Kruskal-Wallis p-value = 3.959e-15) than the proportion of movements that occurred upstream at night (Nemenyi test p-value = 7.2e-05), downstream during day (p-value = 1.1e-08) and upstream during daylight (p-value = 8.6e-14). Total distance moved by individual sea lamprey ranged from at least 1.4 to 1,886.1 laps (20.5 to 28,292.0 m) around the circular artificial stream. Median downstream movement speed (as determined by detection between consecutive antennas) ranged from 8.7 to 24.1 cm·s^-1^ (median: 12.3 cm·s^-1^) among tagged sea lampreys. The number of sea lamprey active during each 24-hr period ranged from 0 to 6 (median 1; Fig 5A) and number of days active for those lamprey detected moving downstream ranged from 1 to 24 (median 3) during the 91 study days. Most activity was nocturnal (Fig 5B) and most initial detections and hence commencement of activity occurred soon after sunset. Little downstream movement occurred during the first three weeks of observation and, of the limited daytime movement, most daytime events occurred during weeks 9–11 of the observation period (Fig 6). Timing of movements, irrespective of date, varied among individuals (Fig 7); the hour of most movement events (mode) occurred during the period 1800–0100 hr for 22 of 25 sea lamprey.

## Discussion

We observed that nearly all downstream migratory activity by juvenile sea lamprey in the artificial and natural streams (net captures and PIT detections) occurred at night and began soon after dark. These results are consistent with observations of other lamprey species (Pacific lamprey, [29]; river lamprey, [39]; pouched lamprey, *G*. *australis*, reviewed in [13]). We are not aware of any empirical estimates of survival during downstream migration of juvenile sea lamprey, but it seems plausible that predation risk could be an important source of mortality during this stage because juvenile lampreys are relatively poor swimmers [40] and migratory habitats typically contain visual predators. In the Columbia River, migratory juvenile Pacific lamprey are the primary prey for northern pikeminnow (*Ptychocheilus oregonesis*) during peak lamprey migration (May and June) while terns and gulls have also been observed preying on downstream migrants [41]. Similarly, nocturnal migration is common for Atlantic salmon smolts and is thought to be a response to avian predation [42].

Although most movement occurred at night, some daytime activity was observed in the artificial stream. Potter [12] speculated that lamprey which had been in the stream throughout the winter had become responsive to migratory stimuli (such as flow) to a degree that movement during high-risk periods (i.e., daytime) was no longer hindered. Likewise, Applegate and Brynildson [16] documented recently metamorphosed sea lamprey moving downstream in daylight during the spring. For the North American landlocked sea lamprey, initiation of metamorphosis typically occurs during July [15] and post-metamorphic juveniles do not feed until reaching the lotic environment [12, 43]. Therefore our study animals may not have fed for about four months at the initiation of the observation period, and about seven months by the conclusion. For fish that cease feeding during migration, sufficient energy reserves are critical for survival [44]. Given the small size of newly metamorphosed sea lamprey (120–160 mm), lipid stores are likely limited and risk of starvation will increase as the season progresses. Starvation can increase the likelihood of undertaking risky behavior and decreased response to environmental cues that indicate danger [45, 46]. Downstream-migrating sea lamprey faced with dwindling energy reserves late in the migratory period could therefore be driven to ignore diel cues that may have previously halted downstream movement. Alternatively, Binder et al. [30] described a reduced response to white light by parasitic sea lamprey relative to larvae. Given that sea lamprey prey are found in open, often clear, environments, reduced sensitivity to light and loss of the avoidance response is likely necessary for finding hosts and thus surviving the parasitic life stage. Future investigations of downstream movement behavior should extend instream observation and monitor condition as well as sensitivity to light relative to migration timing.

The artificial stream study was not designed to test the effect of water temperature, however, relatively little migratory activity during the first 23 days when water temperatures were greater than 4°C. Hanson and Swink [26] observed peak catches of juvenile sea lamprey when water temperatures were near 5°C during the fall and spring, and suggested a possible optimal temperature for initiation of downstream movement. Applegate and Brynildson [16] suggested a threshold response, such that downstream movement began when stream temperatures were less than 6°C. Long-distance migration could be energetically taxing, thus migrating when water temperatures are low would reduce metabolic demands. Unfortunately, water temperature is often associated with both date (day length) and maturation, so controlled studies may be needed to separately assess the effect of variables that are confuounded in observational studies like ours.

Downstream migration by juvenile lampreys likely results from a combination of active and passive behavioral components. Downstream movement for multiple lamprey species is triggered by increased discharge [12,13, 24,25]. Historical observation by Applegate and Brynildson [16] described juvenile sea lamprey movment as passive drifting and the authors speculated that downstream movement of juveniles resulted when lamprey were scoured from the sediment under periods of high discharge. Indeed, the consistent transit times between detection points observed during our instream telemetry study lend support to this description of passive drift. Using water current to move downstream would allow quick transit of long distances and our instream study observations show lamprey to be capable of moving long distances in a relatively short time. However, our observation that downstream movement was only detected during hours of darkness during the in-stream study provides evidence for an active component to downstream migration (i.e., floods also occur during daylight hours). Further, lamprey have been observed to select for high water velocity regions of the stream (river lamprey, [47]; sea lamprey, [33]). Moving during hours of darkness and during flood events (when streams are turbid) could reduce predation mortality, while selecting for times and regions of the stream with increased water velocity could reduce energy expenditure during long-distance migrations for fish species that are considered relatively poor swimmers [40]. Finally, during the artificial stream study we observed a mean movement rate slightly faster than that of the water velocity in the artificial stream, suggesting that at least under low (10–12 cm·s^-1^), stable flow conditions sea lamprey may actively swim downstream. Both active downstream swimming at times and station-holding/drifting within specific stream regions have been described for downstream migrating salmonid smolts [48–51] and seem to be plausible descriptions for downstream movement of juvenile sea lamprey as well.

Our findings are largely observational, but complement a growing body of literature that describes behavior of juvenile lampreys and should be useful for developing management actions and future research questions. The protracted downstream migration by juvenile lampreys [20, 21, 25, 43] may stem from individual variability for initiating downstream movement. That variability makes management difficult for those seeking to limit sources of mortality, such as impingement at hydropower facilities on streams where lamprey are native and imperiled [10, 13], and for managers working to control invasive populations such as the landlocked sea lamprey in the North American Great Lakes [6]. Implementation of seasonal “safe operation” windows for hydropower or water off-takes may not be feasible when downstream movement can occur over 6–8 months. However, if activity is limited to brief, consistent periods after dark [29] then greater potential exists to identify diel periods and stream regions [33, 47] with the highest probabilities of movement and encounter with turbines. Diel operational schedules paired with spatial understanding of juvenile lamprey distribution could simultaneously reduce mortality for imperiled species and allow development of cost-effective capture tools for lamprey where invasive. For example, Moser et al., [10] suggested that if downstream migration is strictly nocturnal, bypass screens, which pose impingement hazards, could potentially be removed at night to allow juvenile lamprey passage when other migratory species are less active. Application of guidance tools such as electric fields [52] might be more feasible if only activated for brief periods during the day or specific locations within a stream channel.

Given the extent of sea lamprey impacts on ecosystems where they are invasive and of human activities on lamprey populations where they are native, it is somewhat surprising that so much remains unknown about the internal (physiological) and external (environmental) processes that influence timing and extent of downstream migration of lampreys. Given that 22 of the 47 lamprey monitored in the artificial stream did not move (upstream or downstream), we assume some migratory cue was lacking which may also account for the variability observed in movement timing. Perhaps the lamprey had not reached some physiological development threshold or a critical abiotic cue was missing to trigger migratory behavior. Our intent was to hold all environmental variables constant to explicitly observed diel differences. Only water temperature changed during the observation period while discharge (velocity and depth), water chemistry, and turbidity remained constant. Observational studies like this one can be useful for generating mechanistic hypotheses [53], but larger-scale controlled experiments and monitoring in natural systems, like the many studies conducted with Atlantic and Pacific salmonids [48–51], may be needed to test such hypotheses.

## Conclusions

Downstream movement of juvenile sea lamprey appears to be initiated shortly after dark and is predominately confined to nocturnal periods. However, individual variability was observed in the initiation of downstream migration, with some lamprey even moving downstream during daylight hours. Evidence exists for both passive drift during juvenile lamprey downstream migration as well as active behavioral components, such as diel behavioral patterns, maintaining station within desirable regions of the stream, or even potential for downstream swimming. Our current observations of downstream migration, paired with recent spatial distribution studies, may allow prediction of encounter probabilities, both temporally and spatially, within a stream, which could aid both conservation and control management efforts.
